# Prevalence and Significance of Patient Prosthesis Mismatch Following Edwards SAPIEN XT and SAPIEN 3 Transcatheter Aortic Valve Replacement

**DOI:** 10.7759/cureus.18044

**Published:** 2021-09-17

**Authors:** Srilakshmi Vallabhaneni, Marsel Matka, Vivek Modi, Matthew Carey, Christopher Sarnoski, Raymond Durkin, Stephen Olenchock, Mehdi Razzaghi, Jamshid Shirani

**Affiliations:** 1 Cardiology, St. Luke's University Health Network, Bethlehem, USA; 2 Internal Medicine, St. Luke's University Health Network, Bethlehem, USA; 3 Interventional Cardiology, St. Luke's University Health Network, Bethlehem, USA; 4 Cardiac Surgery, St. Luke's University Health Network, Bethlehem, USA; 5 Statistics, Bloomsburg University of Pennsylvania, Bloomsburg, USA

**Keywords:** aortic stenosis, transcatheter aortic valve replacement, patient prosthesis mismatch, outcomes, edwards sapien valve

## Abstract

Background

Severe patient prosthesis mismatch (sPPM) after surgical aortic valve replacement is associated with worse outcomes. Limited data exists on the impact of sPPM on outcomes after transcatheter aortic valve replacement (TAVR), especially regarding the newer generation valves. The aim of this study was to evaluate the incidence, determinants, and outcomes of sPPM in patients undergoing TAVR with Edwards SAPIEN XT (ES XT) and Edwards SAPIEN 3 (ES3) valves (Edwards Lifesciences, Irvine, CA, USA).

Methods

We retrospectively reviewed 366 patients who underwent TAVR with ES XT (n = 114) or ES3 (n = 252) valves between July 2012 and June 2018. sPPM was defined as indexed effective orifice area (iEOA) <0.65 cm^2^/m^2^. Kaplan-Meier survival estimates were used to determine outcomes.

Results

Multivariate linear regression analysis was utilized to determine potential independent effects of PPM on outcomes. sPPM was present in 40 (11%) of the patients [8 (7%) ES XT and 32 (13%) ES3] and was associated with female sex, smaller left ventricular outflow tract (LVOT) diameter and aortic valve annular area, absence of prior coronary artery bypass graft (CABG) surgery, shorter height, higher body mass index, and smaller pre-TAVR valve area (all p < 0.05). Among those with ES3 valves, the incidence of sPPM was inversely proportional to the valve size (50%, 25%, 5% and 3% for 20-, 23-, 26- and 29-mm valve sizes, respectively; p < 0.001). At a mean follow-up period of 3.5 ± 1.5 years, there was no difference in all-cause mortality (22.5% vs. 25.6%, p = 0.89) or a composite endpoint of heart failure, arrhythmias, stroke, and myocardial infarction (30% vs. 34%, p = 0.24) in those with or without sPPM.

Conclusion

ES3 was associated with a higher incidence of sPPM, particularly with smaller valve sizes. However, the presence of sPPM as defined by iEOA was not an independent predictor of adverse outcomes in patients undergoing TAVR within an intermediate follow-up period.

## Introduction

Patient prosthesis mismatch (PPM) has been defined as a reduced indexed effective orifice area (iEOA) of an otherwise normally functioning prosthetic valve [1]. Moderate (iEOA between 0.85 cm^2^/m^2^ and 0.65 cm^2^/m^2^) and severe (iEOA < 0.65 cm^2^/m^2^) PPM (sPPM) are reported in 20-70% and 2-20% of patients following surgical aortic valve replacement (SAVR), respectively [2-5]. PPM after SAVR has been shown to adversely affect patients’ outcomes [3-5].

Transcatheter aortic valve replacement (TAVR) has emerged as the intervention of choice for patients with severe symptomatic aortic valve stenosis at high surgical risk [6]. Even with newer generation transcatheter valves and more data to support their use, TAVR is also becoming a potentially viable option for older patients with low to intermediate surgical risk [7]. The reported incidence of PPM after TAVR with earlier generation valves has ranged from 25-45% for moderate and from 1-15% for severe cases [8-10]. This lower incidence, compared to SAVR, has been attributed to imaging-guided preoperative valve sizing, absence of a sewing ring, and a more consistent radial force on the TAVR valve [11]. Additionally, studies of the first-generation TAVR valves have indicated a more benign outcome of PPM in TAVR compared to SAVR [8,11-13]. Significant valvular and para-valvular aortic insufficiency, however, led to relatively unsatisfactory outcomes in the first-generation Edwards SAPIEN and Edwards SAPIEN XT (ES XT) (Edwards Lifesciences, Irvine, CA, USA) balloon-expandable valves [14]. This resulted in the development of the Edwards SAPIEN 3 (ES3) TAVR valve which features an outer annular sealing skirt that significantly reduces paravalvular regurgitation at the expense of EOA. The ES3 valve also has a wider range of available sizes. The specific impact of the newly designed ES3 valve on the incidence of sPPM and its influence on patient outcomes has been evaluated in few studies [15-18]. We aimed to evaluate the incidence, determinants, and clinical outcomes of sPPM in the new generation ES3 compared to the old generation ES XT TAVR valves.

## Materials and methods

Study patients

Institutional review board (IRB) approval was obtained. Consecutive patients with severe symptomatic native valve aortic stenosis who had successfully undergone TAVR with ES XT or ES3 prostheses between July 2012 and June 2018 were identified. Patients with severe symptomatic aortic stenosis were considered candidates for TAVR if they had a high Society of Thoracic Surgeons (STS) Risk Score or if surgery was deemed high risk due to comorbidities. Patient eligibility for TAVR was based on the consensus of a multidisciplinary heart team that included cardiologists, interventional cardiologists, and cardiac surgeons. The prosthesis size was selected according to the average annular diameter and area measured on pre-procedural transesophageal echocardiography and/or multidetector computed tomography. The valves were delivered via the transfemoral or transapical approaches. Patients with valve-in-valve interventions or suboptimal echocardiographic images were excluded from the study. A total of 409 patients underwent TAVR with ES XT or ES3 valves during this time period. After excluding 25 patients who underwent valve-in-valve interventions, the final sample size was 384 patients. Of these, 366 patients who had pre-discharge transthoracic echocardiography (TTE) and follow-up data available were included in the final analysis of clinical outcomes and predictors of sPPM.

Transthoracic echocardiography

TTE was performed at baseline and before discharge following TAVR by experienced sonographers using commercially available ultrasound machines [GE Vivid E9 (GE Medical Systems, Milwaukee, WI, USA) or Philips Epiq 7 (Philips Healthcare, Andover, MA, USA)]. A comprehensive study that included two-dimensional B-mode as well as a pulse-wave, continuous-wave, and color Doppler was performed according to current recommendations [19]. The aortic valve effective orifice area (EOA) was calculated using the continuity equation and was indexed to the body surface area (iEOA). All TTE measurements were made offline and were averaged from three consecutive sinus rhythm beats or five consecutive beats in patients with atrial fibrillation. Post-TAVR paravalvular aortic regurgitation (AR) was evaluated using color Doppler and was semi-quantitatively graded as mild (1+), moderate (2+), moderately severe (3+), or severe (4+).

Definition of PPM

The presence of PPM was assessed on post-procedural day 1, pre-discharge TTE by calculating iEOA using the continuity equation. It was defined as moderate if iEOA was between 0.65 and 0.85 cm^2^/m^2^ and as severe if iEOA was <0.65 cm^2^/m^2^ [2,20].

Endpoints

The primary endpoints were all-cause mortality and a composite endpoint of acute myocardial infarction (AMI), arrhythmias (new-onset atrial fibrillation or flutter, sustained ventricular tachycardia or ventricular fibrillation), acute decompensated heart failure or stroke. Outcomes were evaluated after stratifying patients based on the presence or absence of PPM.

Statistical analyses

Continuous variables were expressed as mean ± SD. Categorical variables were presented as absolute numbers or percentages. For continuous variables, significant differences between groups were analyzed using Student’s t-test or Fisher’s exact test. χ2 test was used to assess the differences between categorical variables. Univariate and multivariate linear regression analyses were performed to determine predictors of post-TAVR sPPM. A statistically significant difference was defined as a p < 0.05. Cumulative survival rates were analyzed using the Kaplan-Meier method. The differences were then assessed using the log-rank test.

## Results

Demographic and clinical information

Table 1 summarizes the demographic and clinical characteristics of the patients who underwent TAVR (n = 366) and compares the findings in those with ES XT (n = 114) or ES3 (n = 252) valves. The average age of the patients was 82 ± 7 years with a high prevalence of atherosclerotic risk factors. Significant differences existed between those with the ES XT compared to the ES3 valves. Patients who received the ES3 valve were younger (81 ± 7 vs. 83 ± 7, p = 0.005), more often smokers, had significantly lower Society of Thoracic Surgeons risk score (5.8 ± 2.8 vs. 8.4 ± 3.8, p < 0.0001) and a significantly higher prevalence of hypertension, coronary artery disease, cerebrovascular accident, hyperlipidemia, and chronic kidney disease. The prevalence of atrial fibrillation was higher in those undergoing TAVR with the ES XT valve.

**Table 1:** Baseline demographic and clinical characteristics of patients undergoing transcatheter aortic valve replacement (TAVR) with Edwards SAPIEN XT (ES XT) and Edwards SAPIEN 3 (ES3) Valves STS: Society of Thoracic Surgeons; NS: Not significant.

		All patients (n = 366)	ES XT (n = 114)	ES3 (n = 252)	p-value
Age (years)		82±7	83±7	81±7	0.005
Men (%)		170 (46)	53 (46)	117 (46)	NS
White race (%)		354 (97)	111 (97)	243 (96)	NS
Height (in)		65±4	65±4	65±4	NS
Weight (lbs)		181±44	182±43	181±45	NS
Body surface area (m^2^)		1.9±0.3	1.9±0.2	1.9±0.3	NS
STS Risk Score (%)		6.6±3.3	8.4±3.8	5.8±2.8	<0.0001
Hypertension (%)		358 (98)	107 (94)	251 (99.6)	<0.001
Coronary artery disease (%)		268 (73)	71 (62)	197 (78)	<0.001
Coronary artery surgery (%)		85 (23)	28 (25)	57 (23)	NS
Prior permanent pacemaker or defibrillator (%)		66 (18)	22 (19)	44 (17)	NS
Chronic kidney disease (%)		175 (48)	43 (38)	132 (52)	0.009
Smoker (%)		171 (47)	28 (25)	143 (57)	<0.001
Diabetes mellitus (%)		79 (22)	24 (21)	55 (22)	NS
Cerebrovascular accident (%)		48 (13)	1 (0.01)	47 (19)	<0.0001
Peripheral artery disease (%)		45 (12)	19 (17)	26 (10)	NS
Carotid stenosis	Moderate	52 (14)	17 (15)	35 (14)	NS
	Severe	21 (6)	3 (3)	18 (7)	NS
Atrial fibrillation (%)		157 (43)	58 (51)	99 (39)	0.038
Hyperlipidemia (%)		325 (89)	92 (81)	233 (92)	<0.001
Pre-TAVR coronary stenting		38 (10)	7 (6)	31 (12)	NS
Hemoglobin (gm/dL)		12.4±4.6	12.1±1.7	12.5±5.5	NS
Hematocrit (%)		37.1±4.9	37.2±4.5	37.1±5.1	NS
Platelets (1000/µl)		210.8±78.6	213.4±80.7	209.6±77.8	NS

Echocardiographic and computed tomography data

Table 2 summarizes baseline and one-day post-procedural echocardiographic parameters in patients undergoing TAVR. The mean left ventricular (LV) ejection fraction was 56.3% and was preserved (≥55%) in 278 (76%) of the patients. Calculated aortic valve area (AVA) using the continuity equation was 0.8 cm^2^ at baseline and 63% of the patients had a mild or greater degree of aortic insufficiency. When compared to ES3, those who underwent ES XT TAVR had smaller AVA (0.68±0.20 vs. 0.80±0.30 cm^2^; p = 0.0003) at baseline and higher prevalence of pre-procedural mild or greater degrees of aortic insufficiency (78% vs. 56%, p = 0.001). On post-procedural day 1, the peak and mean trans-prosthetic velocities and gradients were significantly higher in ES3 patients compared to ES XT, while the incidence of a mild or greater degree of aortic insufficiency was higher in ES XT patients (55% vs. 30%, p < 0.0001). Overall, sPPM was observed in 40 (11%) of the patient population with a trend towards a higher proportion of patients with sPPM among the ES3 group (p = 0.10). The aortic valve annular area was 477.2 ± 113.7 mm^2^ by computed tomography and was significantly greater in patients with ES3 compared to those with ES XT (488.3 ± 119.1 vs. 451.6 ± 95.6 mm^2^, p = 0.002).

**Table 2:** Baseline and post-procedural day 1 echocardiographic findings in patients undergoing transcatheter aortic valve replacement with Edwards SAPIEN XT (ES XT) and Edwards SAPIEN 3 (ES3) valves NS: Not significant

		Total (n = 366)	ES XT (n = 114)	ES 3 (n = 252)	p-value
Pre-procedural findings					
Left ventricular ejection fraction (%)		56.3±11.1	56.8±10.5	56.3±11.4	NS
Left ventricular ejection fraction ≥55%		278 (76%)	88 (77%)	190 (75%)	NS
Left ventricular ejection fraction <55%		88 (24%)	26 (23%)	62 (25%)	NS
Aortic valve area by continuity equation (cm^2^)		0.87±0.3	0.68±0.2	0.80±0.3	0.0003
Aortic regurgitation (≥mild)		231 (63%)	89 (78%)	142 (56%)	0.001
Post-procedural day 1 findings					
Left ventricular ejection fraction (%)		58.8±10.7	58.7±10.7	58.8±10.7	NS
Aortic valve	Peak velocity (cm/s)	207.9±47.2	187.8±34.3	217±49.6	<0.0001
	Mean velocity (cm/s)	141.4±32.5	126.1±23.1	148.3±33.8	<0.0001
	Peak gradient (mmHg)	18.2±8.1	14.6±5.3	19.8±8.6	<0.0001
	Mean gradient (mmHg)	9.3±4.4	7.1±2.7	10.2±4.7	<0.0001
	Insufficiency (≥Mild)	145 (38%)	68 (55%)	77 (30%)	<0.0001
	Area by continuity equation (cm^2^)	1.8±0.52	1.84±0.51	1.78±0.52	NS
	Severe patient prosthesis mismatch	40 (11%)	8 (7%)	32 (13%)	0.1

Comparison of baseline findings in patients with or without sPPM

As shown in Table 3, significant differences existed in baseline characteristics between patients with (n = 40) and without (n = 326) sPPM following TAVR. Those with sPPM were found to be more often women (77% vs. 50%, p = 0.001) of shorter height (63.7 ± 3.7 vs. 65.2 ± 4.2, p = 0.03) and a higher body mass index (33.3 ± 9.2 vs. 29.6 ± 6.4, p = 0.0025). Patients with sPPM had a smaller aortic valve annular area by computed tomography when compared to those without sPPM (435.1 ± 110.1 vs. 483 ± 113.8, p = 0.0086). A history of prior coronary artery bypass surgery was less often present in those with sPPM (8% vs. 25%, p = 0.012). All other parameters were similar between the two groups. Table 4 compares the echocardiographic findings in those with and without sPPM. Patients with sPPM had significantly smaller left ventricular outflow tract (LVOT) diameters (18.9 ± 1.7 vs. 20.1 ± 1.7 mm, p=<0.0001) as well as a lower aortic valve area prior to TAVR and iEOA after TAVR. On average, patients with sPPM had a post-procedural iEOA that was smaller than those without sPPM by 0.42 cm^2^/m^2^.

**Table 3:** Baseline characteristics of patients with or without severe patient prosthesis mismatch NS: Not significant; TAVR: Transcatheter aortic valve replacement.

Patient prosthesis mismatch		Present (n = 40)	Absent (n = 326)	p-value
Age (years)		81.1±8.4	81.9±7.1	NS
Men (%)		9 (23)	163 (50)	0.001
White race (%)		40 (100)	316 (96)	NS
Height (in)		63.7±3.7	65.2±4.2	0.03
Weight (lbs)		190.5±53.6	179.7±42.9	NS
Body mass index (kg/m^2^)		33.3±9.2	29.6±6.4	0.0025
Body surface area (m^2^)		1.9±0.3	1.9±0.2	NS
Society of Thoracic Surgeons risk score (%)		6.9±3	6.6±3.3	NS
Hypertension (%)		40 (100)	320 (98)	NS
Coronary artery disease (%)		30 (75)	240 (73)	NS
Coronary artery surgery (%)		3 (8)	83 (25)	0.012
Prior permanent pacemaker or defibrillator (%)		10 (25)	57 (17)	NS
Chronic kidney disease (%)		22 (55)	155 (47)	NS
Smoker (%)		20 (50)	151 (46)	NS
Diabetes mellitus (%)		11 (28)	68 (21)	NS
Cerebrovascular accident (%)		8 (20)	40 (12)	NS
Peripheral artery disease (%)		4 (10)	41 (13)	NS
Carotid disease	Moderate	7 (17.5)	45 (14)	NS
	Severe	1 (2.5)	20 (6)	NS
Atrial fibrillation (%)		19 (48)	140 (43)	NS
Hyperlipidemia (%)		34 (85)	292 (89)	NS
Pre-TAVR coronary stenting		6 (15)	36 (11)	NS
Hemoglobin (gm/dL)		11.5±1.7	12.5±4.9	NS
Hematocrit (%)		35.9±4.8	37.3±4.9	NS
Platelets (1000/µl)		218.2±92.9	209.5±76.5	NS
Aortic valve annular area on CT (mm^2^)		435.1±110.1	483±113.8	0.0086
Transfemoral approach		34 (85)	281 (86)	NS
Edwards SAPIEN XT		8 (20)	106 (33)	NS
Edwards SAPIEN 3		32 (80)	220 (67)	NS

**Table 4:** Baseline and post-procedural day 1 echocardiographic findings in patients with or without severe patient prosthesis mismatch NS: Not significant

Severe patient prosthesis mismatch		Present (n = 40)	Absent (n = 326)	p-value
Pre-procedural findings				
Left ventricular ejection fraction (%)		56.9±10.9	56.2±11.2	NS
Left ventricular ejection fraction ≥55%		32 (80%)	246 (75%)	NS
Left ventricular ejection fraction <55%		8 (20%)	82 (25%)	NS
Aortic valve area by continuity equation (cm^2^)		0.7±0.2	0.8±0.3	0.007
Aortic regurgitation (≥mild)		28 (70%)	204 (62%)	NS
Left ventricular outflow tract diameter (mm)		18.9±1.7	20.1±1.7	<0.0001
Post-procedural Day 1 findings				
Left ventricular ejection fraction (%)		57.9±9.6	58.8±10.8	NS
Aortic valve	Peak velocity (cm/s)	254.2±54	203±41.2	<0.0001
	Mean velocity (cm/s)	173.4±36.1	137.4±29.6	<0.0001
	Peak gradient (mmHg)	51.9±12	40±9.6	<0.0001
	Mean gradient (mmHg)	26.2±10.7	17.2±7.1	<0.0001
	Insufficiency (≥mild)	15 (38%)	132 (40%)	NS
	Effective orifice area (cm^2^)	1.1±0.2	1.9±0.5	<0.0001
	Indexed effective orifice area (cm^2^/m^2^)	0.56±0.07	0.98±0.25	<0.0001

Predictors of sPPM

**Figure 1:**
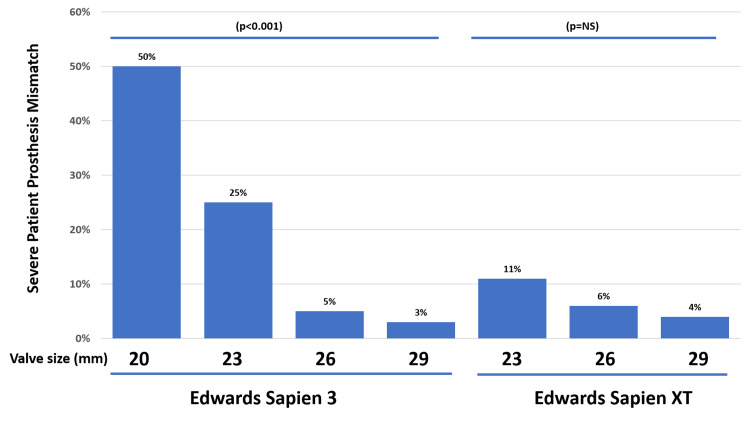
Bar graphs indicating a prevalence of severe patient prosthesis mismatch among Edwards SAPIEN XT and three valves of different sizes. Smaller Edwards SAPIEN 3 valves had a particularly higher prevalence of severe patient prosthesis mismatch.

sPPM was associated with female sex (77% vs. 50%, p = 0.001), a smaller left ventricular (LV) outflow tract diameter (18.9±1.7 vs. 20.3±1.7, p < 0.0001), a larger body mass index (33.3±9.2 vs. 29.6±6.4, p < 0.0025) and a lower prevalence of previous coronary bypass surgery (8% vs. 25%, p = 0.012). LV ejection fraction (57±11% vs. 56±11%), body surface area (1.9±0.3 vs. 1.9±0.2 m^2^) and age (81±8 vs. 82±7 years) were not statistically significantly different between those with or without sPPM (all p > 0.05) (Tables 3, 4). Valve size was predictive of sPPM only among those with ES3 valves and not among ES XT group. Among ES3 patients, the incidence of sPPM was 50% (n = 5) for 20 mm, 25% (n = 20) for 23 mm, 5% (n = 5) for 26 mm, and 3% (n = 2) for the 29 mm valve (p < 0.001) (Figure 1).

Clinical outcomes

**Figure 2:**
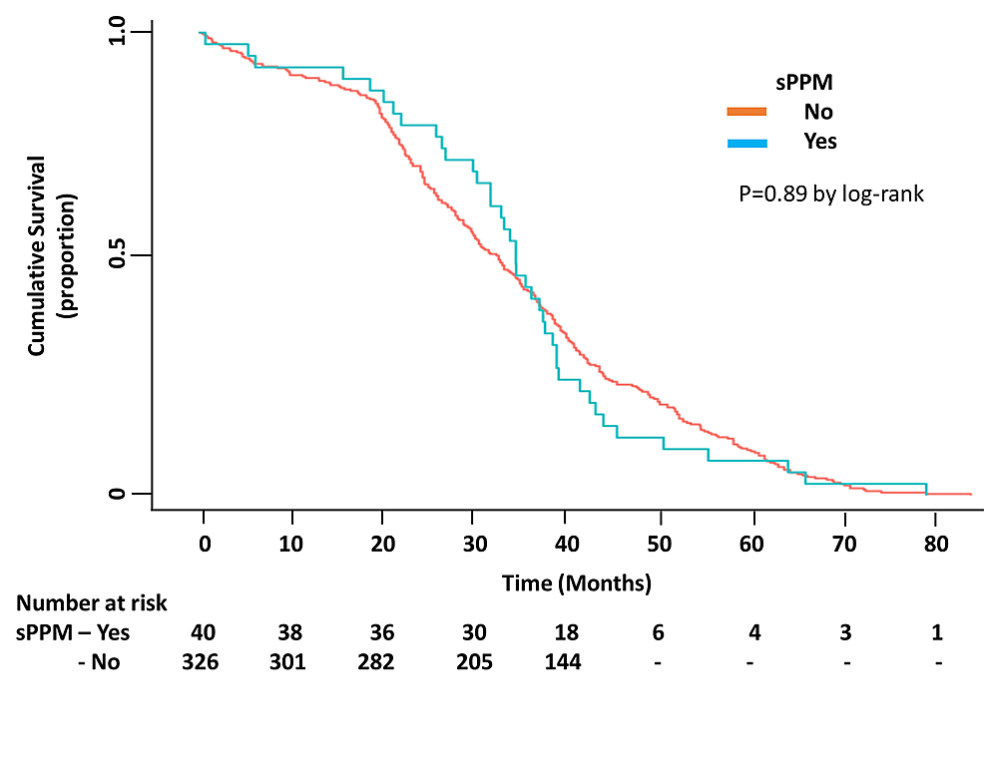
Kaplan-Meier curve showing no significant difference in all-cause mortality among patients with and without severe patient prosthesis mismatch (sPPM)

**Figure 3:**
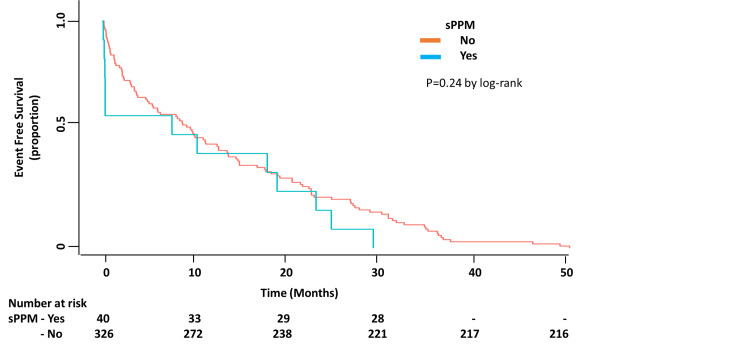
Kaplan-Meier curve showing no significant difference in a composite endpoint of acute decompensated heart failure, acute myocardial infarction, arrhythmias and stroke among patients with and without severe patient prosthesis mismatch (sPPM)

At a mean follow-up of 3.5±1.5 years, a total of 97 patients died, of these, nine (9.3%) had sPPM. There was no difference in the prevalence of sPPM among the survivors and the deceased (11.5% vs. 9.3%, p = NS) (Figure 2). All-cause mortality was similar among patients with or without sPPM (22.5% vs 26.9%, p = NS). Only 25 of the 97 deaths were determined to have been due to a cardiovascular cause. Of those 25 cardiovascular deaths, only one occurred in a patient who had sPPM. Over the duration of follow-up, there was no significant difference in the incidence of hospitalization for a composite endpoint of stroke, arrhythmias, heart failure, or acute myocardial infarction among patients with or without sPPM (30% vs. 34%, p = NS) (Figure 3).

## Discussion

Findings of the present study

The main findings of the present study were: (1) a trend existed towards higher prevalence of sPPM among ES3 compared to ES XT valves (13% vs. 7%, p = 0.10); (2) among those with ES3 valves, a significantly higher proportion of smaller size valves (20 and 23 mm) were associated with sPPM (28% vs. 4%, p < 0.0001); and (3) the higher prevalence of sPPM in patients with ES3 valves was not associated with an increased rate of adverse outcomes over the follow-up period of 3.5 ± 1.5 years. The predictors of sPPM in our study were female sex, a smaller LVOT diameter, a smaller aortic valve annular area, a lower prevalence of prior coronary artery surgery, shorter height, higher body mass index, and a smaller pre-TAVR aortic valve area. Pre-procedural aortic regurgitation (≥mild) was not associated with sPPM.

ES XT and ES3 valves

The ES XT valve was first implanted in our institution in 2012. The valve had already undergone revisions at that time. The current model, released in 2010, had a balloon-expandable cobalt-chromium alloy frame with bovine pericardium cusps and was available in 23, 26, and later in 29 mm sizes. Among 114 patients who underwent TAVR with ES XT valve in our institution, sPPM occurred in eight patients (7%) and another seven patients (6%) had significant (at least moderate) post-procedural aortic insufficiency. In August 2015, we started to use the newer generation ES3 valve that aimed to reduce the incidence of significant paravalvular aortic insufficiency by adding an outer polyethylene terephthalate skirt [21]. The valve was eventually available in 20, 23, 26- and 29-mm sizes. Among the 252 patients who underwent TAVR with the ES3 valve at our institution, four (1.6%) patients had post-procedural moderate or worse aortic insufficiency. The incidence of sPPM, however, was noted to be greater than what was observed with the ES XT valve (13% vs. 7%, p = 0.10). The latter was felt to be due to the new design and the introduction of the smaller valve size (i.e., 20 mm). Previous studies had also shown that smaller ES3 valves are associated with a higher incidence of sPPM [10, 16-18, 22]. In our study, statistically significant differences were noted between ES XT and ES3 with regards to peak (14.6 ± 5.3 vs. 19.8 ± 8.6 mmHg, p < 0.0001) and mean (7.1 ± 2.7 vs. 10.2 ± 4.7 mmHg, p < 0.0001) trans-prosthetic gradients as well as the dimensionless index (0.57 ± 0.14 vs. 0.52 ± 0.12, p = 0.0008). A comprehensive review of the data from the PARTNER trial has also shown slightly lower EOA, higher mean gradients, and lower dimensionless valve index values for ES3 as compared to ES XT [23]. The overall incidence of sPPM in our cohort was 11%. This is comparable to the reported incidence of 1-28% in recent studies [8,10,13,16,17,24]. We did observe a trend towards a higher incidence of sPPM among patients with ES3 compared to those with ES XT (12% vs. 6%) as shown in other studies [16,17]. Previous studies have also shown that smaller ES3 valves are associated with a higher incidence of sPPM [10,16-18,22].

Predictors of sPPM

A higher incidence of post-TAVR PPM in female patients has been previously reported [25]. It has also been demonstrated that a smaller LVOT may be predictive of PPM for the CoreValve prosthesis (Medtronic, Minneapolis, MN, USA) [13,26]. Contrary to the finding of this study, several reports have shown a direct correlation between prior history of coronary artery disease or CABG with a higher incidence of PPM in patients undergoing TAVR [9,10,16]. Contrary to our cohort, taller rather than shorter stature has been shown to predict PPM [9,16]. Finally, a smaller pre-TAVR aortic valve area has been found to be predictive of PPM in several prior studies [9,10,13,16].

In a large registry, primarily of older generations TAVR valves, smaller valve size (≤23 mm in diameter), valve-in-valve procedure, larger body size, lower left ventricular ejection fraction, non-white/Hispanic race, female gender, and younger age were identified as independent predictors of PPM [10]. A Japanese registry has demonstrated a very low prevalence (0.7%) of sPPM following TAVR and has identified younger age, larger body size, smaller pre-TAVR aortic valve area, smaller annular area, and use of the ES3 valve as independent predictors of sPPM [16]. Several studies have demonstrated a higher incidence of sPPM in the newer generation TAVR valves [17,18].

Outcomes

Studies on SAVR populations have demonstrated that sPPM adversely affects long-term survival and has an incidence ranging between 20 and 70% [4,5,27]. Several studies have examined short- and long-term outcomes of post-TAVR PPM with mixed results. In these studies, the incidence of PPM has ranged between 1-28% [8, 10, 13, 15, 16, 23, 25]. One study has reported lower mortality in TAVR patients with PPM (iEOA <0.85 cm^2^/m^2^) [11]. Others have shown no differences in mortality, major adverse cardiac events, or symptomatic improvement among patients with or without post-TAVR PPM with the older generation valves at six months [13]. A Japanese multicenter registry of ES XT, ES3, or CoreValve prostheses found no impact of PPM on mortality at one year [16]. However, the largest study to date, including 62,125 patients, has indicated that sPPM is associated with higher mortality and heart failure hospitalizations at one year [10]. The differences in the latter two studies are probably explained by significantly different body sizes and incidence of PPM [10,16]. Our study is unique in that we examined only patients undergoing TAVR with ES XT and ES3 prostheses. We found no difference in all cause-mortality or in a composite endpoint of hospitalization for heart failure, myocardial infarction, arrhythmias and stroke at a mean follow-up period of 3.5 years in patients with or without post-TAVR sPPM. The original focus of TAVR was on the quality of life rather than longevity. With the expansion of the indications for TAVR to younger, lower-risk individuals, longevity will become increasingly important. It has been shown that patients with PPM do have decreased quality of life at six months following TAVR [10].

Limitations

This study was retrospective, non-randomized and sequential. As a result, all inherent limitations of such a design are applicable to the findings. Although our study included all-cause mortality and defined a composite endpoint, other outcomes such as quality of life or left ventricular reverse remodeling were not examined. Mortality data can be underestimated as we relied on electronic medical records and publicly available obituary reports. We studied only the Edwards SAPIEN valves so the findings may not be extendable to other TAVR valves already in use. Large, prospective, randomized controlled, multicenter trials are needed to validate our findings. Finally, methodologic aspects of determining PPM are still being debated and the prevalence and clinical significance of PPM have been inconsistent for both TAVR and SAVR prostheses. Nevertheless, the present study shows that the third generation ES3 valve reduces post-procedural aortic regurgitation without adversely affecting intermediate-term outcomes despite higher average trans-prosthetic velocities and gradients.

## Conclusions

sPPM, as defined by iEOA, was associated with female sex, smaller LVOT diameter and aortic valve annular area, lower pre-TAVR AVA, lower incidence of prior CABG, shorter stature, and higher BMI with a higher incidence in smaller size ES3 valves. There was no difference in all-cause mortality or a composite endpoint of heart failure, arrhythmias, stroke, and myocardial infarction at a mean follow-up period of 3.5 years among those with or without sPPM.
